# Editorial: Comprehensive strategies for public health education across diverse audiences and settings to control nosocomial infection

**DOI:** 10.3389/fpubh.2026.1802790

**Published:** 2026-03-04

**Authors:** Mingke Wang, Yingchun Xiang, Chunhui Li, Mohan Bairwa, Alfonso J. Rodriguez-Morales

**Affiliations:** 1 Naval Medical Center of Chinese People's Liberation Army, Naval Medical University, Shanghai, China; 2 Department of Nursing, School of International Medical Technology, Shanghai Sanda University, Shanghai, China; 3 Department of Infection Control Center, Xiangya Hospital, Central South University, Changsha, China; 4 Centre for Community Medicine, All India Institute of Medical Sciences, New Delhi, India; 5 Faculty of Health Sciences, Universidad Científica del Sur, Lima, Peru; 6 Grupo de Investigación Biomedicina, Facultad de Medicina, Fundación Universitaria Autónoma de las Américas-Institución Universitaria Visión de las Américas, Pereira, Colombia

**Keywords:** community health education, effects evaluation, hospital health education, methods, nosocomial infection, prevention, public health education, strategies

Management of nosocomial infections (NIs), alternatively termed healthcare-associated infections (HAIs), is crucial for maintaining high standards of medical quality (1). The implementation of targeted health education programs in hospitals not only improves medical quality and patient outcomes but also enhances the overall therapeutic environment (2, 3). Such initiatives are essential for fostering harmonious relationships between healthcare providers and patients, increasing patient satisfaction, and safeguarding the mental and physical wellbeing of medical staff. Furthermore, continuing education and legal training for healthcare personnel on NIs can strengthen their legal awareness and enhance their capacity to comply with healthcare regulations (4, 5). The international research community has been increasingly focused on the role of public health education in managing NIs across diverse populations and settings. Despite significant emerging research, numerous challenges remain in effectively tailoring health education and in evaluating its impact across different demographics, time points, locations, and diseases (6). Accordingly, this Research Topic was proposed to compile a collection of manuscripts that explore recent advances and provide fresh perspectives on the comprehensive strategies, methodologies, theoretical frameworks, content, and evaluation approaches of public health education tailored to NIs control. In total, 13 papers have been accepted for this Research Topic, including two systematic reviews and 11 original research articles.

Enhancing the cleaning and disinfection of medical equipment can substantially reduce HAIs and improve patient outcomes (7). To compare the efficacy of manual cleaning, alkaline multi-enzyme immersion combined with ultrasonic cleaning, and automatic reprocessing machines in laparoscope decontamination, Li et al. conducted a systematic review and meta-analysis of 11 randomized controlled trials involving 4,661 cases. Their findings indicated that enhanced cleaning methods—alkaline multi-enzyme cleaning with ultrasonic assistance or automated reprocessing—yield modest improvements in laparoscope decontamination compared to manual cleaning alone. Nevertheless, they emphasized that optimal cleaning protocols for clinical use require not only appropriate equipment but also comprehensive staff training, robust quality-monitoring systems, and alignment with local infection-prevention priorities.

Surgical site infections (SSIs) in patients have a significant negative impact on patient recovery and their healthcare expenditures, which also have the highest prevalence among infection types of NIs in low- and middle-income countries (8, 9). Although the incidence of SSIs has reduced to a certain extent with the advancement in aseptic technique and optimization of perioperative antibiotic management in recent years, a comprehensive understanding of their risk factors, as well as proactive preventive and prophylactic strategies, is still imperative (9, 10). Ma et al. reported that clinicians demonstrated lower accuracy in implementing waterless surgical hand antisepsis (WSHA) guidelines before the intervention. Although the WSHA score increased with the implementation of a tailored, multifaceted improvement strategy, it still fell short of the mid-term assessment score. Therefore, continuous supervision and training for WSHA, raising awareness of hand disinfection, and promoting a culture of hand hygiene remain necessary.

Hospitals harbor a substantial number of confirmed coronavirus disease 2019 (COVID-19) patients and asymptomatic carriers, accompanied by high population density and a larger susceptible population, which may serve as potential triggers for NIs during the COVID-19 pandemic (11). Practical experiences in hospitals could provide significant assistance to the prevention, control, and clinical practice of COVID-19 in all countries worldwide (12). Petkar et al. described the epidemiology and management of their first nosocomial healthcare worker (HCW) outbreak in their hospital, reviewed their interventions and investigation findings, highlighted their lessons learned and strategies developed to tackle poor compliance, including social and behavioral factors, and concluded that addressing non-compliance with behavioral and social infection control measures was a critical component in management of HCW COVID-19 outbreaks. Their multi-pronged infection prevention and control (IPC) precautions focusing on strict compliance with social distancing, appropriate use of personal protective equipment (PPE), rigorous hand hygiene, and effective environmental cleaning, and taking into consideration the local institutional culture Good leadership, a culture of speaking up for patient safety and measures to link individual HCW IPC practices to their annual professional practice evaluations could also be effective measures in other similar NIs outbreak situations.

Nurses can prevent NIs by different methods, such as appropriate hand hygiene, skin antisepsis, wearing gloves and masks, using replacement infusion sets, isolating infected patients, applying the principles of standard precautions, preventing accidental hand contacts with needles, and having significant roles in increasing awareness on infection control issues and motivating staff to improve IPC practice. However, low levels of knowledge and poor performance in infection control among nurses underscore the need for education (13). In this Research Topic, An et al. constructed a set of standardized nursing staff refresher training systems in traditional Chinese medicine (TCM) hospitals using the Delphi method, and evaluated its application effect, which also provided a reference basis for optimizing the content of nursing training, improving their professional quality and skills in NIs prevention and control practices.

To explore the clinical value of PDCA circulation nursing in nursing management of NIs, Wang et al. investigated the effect of application of PDCA cycle in hospital-acquired infection nursing management with comparing the nursing satisfaction, nosocomial infection rate, nursing work quality score, patient self-management ability, medical equipment and goods qualification rate and nurse professional skill score by selecting 120 inpatients in a hospital in China. Their results demonstrated that the PDCA cycle could improve nursing quality, enhance patient self-management and satisfaction, and significantly reduce the incidence of NIs in clinical practice. They may prove to be a valuable management strategy in nosocomial infection control in the future.

To assess the efficacy of World Health Organization (WHO) multimodal strategy on healthcare workers' knowledge and compliance on hand hygiene (HH), Hannachi et al. conducted a quasi-experimental study with a pre- and post-intervention design in a teaching hospital in Tunisia, and found that knowledge of major causes responsible for healthcare associated infections, awareness of recommended duration of Alcohol-based hand rub or soap and water, and the global rate of HH compliance increased. In contrast, the HH compliance rate with optimal respect of the prerequisites decreased significantly. Their study indicated that the WHO multimodal strategy could increase the HH compliance rate, but no significant improvement in HCWs' knowledge was observed.

In another study, Ramos-Meza et al. adapted and validated the HAInnovPrev scale, a tool for assessing the prevention and control of healthcare-associated infections (HAIs) among nursing students utilizing an exploratory factor analysis approach. One of the main advantages of this modified scale is its ability to reduce the complexity of the original 15-dimensional instrument into 11 refined dimensions while encompassing key aspects such as institutional compliance, personal motivation, educational influences, academic resources, career commitment, emotional exhaustion, and adherence to hygiene protocols. This development process may improve its practical applicability and clarity, and would be beneficial for educational and clinical applications.

Additionally, Hu et al. evaluated the effectiveness of the Direct Observation of Procedural Skills (DOPS) method in enhancing hospital infection control training by comparing theory test scores and satisfaction ratings between 196 infection control staff from clinical departments, who were divided into a control group and an experimental group. They found that applying the DOPS method in infection control training could not only improve trainees' practical infection control skills but also increase knowledge retention and participant satisfaction, making DOPS a promising targeted intervention for broader implementation in clinical infection control education.

Moreover, Chen et al. assessed the effectiveness of the new conceive-design-implement-operate (CDIO) model and traditional lecture-based learning (LBL) on nursing students' HAIs learning outcomes through comparing their pre-and post-training test scores, collecting the students' feedback on the course and teaching by the Course Experience Questionnaire (CEQ), and found that applying the CDIO framework to nursing education in HAIs courses could enhance nursing students' practical application skills and particularly promote their sustained retention in this area. Their results indicated that the CDIO teaching model had significant advantages in enhancing the course experience and teaching effectiveness, including improvements in effective teaching, clear goals and standards, appropriate assessment, generic skills, and an emphasis on independence, compared with the control group; however, a significant increase in workload was also reported.

Previous studies have identified that immigrants have insufficient health education and basic health literacy, highlighting the importance of strengthening health education in improving their health (14, 15). In the study reported by Xu et al., the authors investigated segmented assimilation theory in health education utilization (HEU) and its influencing factors among internal migrant populations in China, explored diverse assimilation patterns among internal migrants in four clusters: first-generation classic assimilation, first-generation integration assimilation, second-generation segmented assimilation, and second-generation underclass assimilation, found that factors such as ethnicity, marital status, employment status, educational attainment, hukou type, health insurance type, time of access to healthcare, social integration, social participation, establishment of health records, and issues encountered in host and origin places significantly influenced their HEU, underscored the need for targeted policy interventions addressing those diverse migrant assimilation patterns, and indicated that comprehensive health management strategies for migrant populations, including diverse health education programs, should be developed.

Catheter-associated urinary tract infection (CAUTI) is one of the most common HAIs in clinical practice (16). The knowledge, attitudes, and practices (KAP) of healthcare workers play an essential role in infection prevention and control; however, studies on factors affecting KAP in obstetrics and gynecology remain limited. In the study reported by Qu et al., the authors investigated the KAP scores of healthcare workers in an OB/GYN hospital regarding CAUTI prevention. They identified the influencing factors, including gender, educational level, department, professional role (doctor vs. nurse), years of service, clinical instructor, leadership emphasis, and training frequency, which offered data that can inform effective CAUTI prevention strategies. They provided evidence to improve targeted training programs and optimize frontline infection-control measures.

Effective fecal incontinence (FI) management is critical not only for patient wellbeing but also for preventing HAIs (17, 18). Yu et al. investigated the real-world use and challenges of FI collection products for long-term, bed-bound hospital patients, drawing on caregivers' (nurses, nursing assistants, and family members) insights, and found that disparities in FI product use stem mainly from economic constraints, training gaps, and limited awareness. Their results indicated that enhancing caregiver training, streamlining product distribution, and broadening insurance coverage could strengthen FI management, reduce the incidence of incontinence-associated dermatitis, improve patient outcomes, and reduce HAIs, providing guidance for infection control policy and practice.

Occupation-related respiratory diseases (ORRD) among sanitary workers (SWs), including hospital cleaners who play a critical role in IPC for housekeeping, environmental cleaning, and waste management within hospitals, have been a significant public health challenge (19). However, few studies have quantified ORRD among these groups. Tolera et al., estimated the pooled prevalence of ORRD among SWs globally using a systematic review and meta-analysis, revealed that the pooled prevalence among all SWs was 32.56% (95%CI: 25.78, 39.34%) worldwide, and found that these illnesses were more prevalent in low-income countries than in high-income countries, due to risk factors such as inadequate PPE, lack of screening, insufficient occupational health and safety procedures, lack of supervision and training, lack of commitment, and lack of focus on workplace risk by sector leaders. Their results indicated that government policy changes and other preventive measures, such as providing access to protective equipment, offering occupational health and safety training, and ensuring regular supervision, are required, particularly in low-income countries.

Overall, our previous Research Topic has shown that COVID-19 exerted an extensive impact on NIs, highlighting that the NIs control system must continue to evolve and improve even in the post-COVID-19 era (20). Public health education across diverse audiences and settings is essential for effective IPC programs (21, 22). Accordingly, this Research Topic collected and published 13 papers about comprehensive strategies for public health education across diverse audiences and settings, including education for patients, medical staff, interns, and the community at various stages of the healthcare journey, from admission through post-discharge, and across various medical specialties. In the future, with the advancements in health technologies such as e-health, Artificial Intelligence, and the Internet of Things, coupled with global trends including increased population mobility, aging demographics, and augmenting drug resistance, further research into comprehensive public health education strategies for reducing HAIs remains imperative (23, 24).
